# A quantum dot-based lateral flow immunoassay for the rapid, quantitative, and sensitive detection of specific IgE for mite allergens in sera from patients with allergic rhinitis

**DOI:** 10.1007/s00216-020-02422-0

**Published:** 2020-02-12

**Authors:** Zheng-Yan Liang, Yu-Qin Deng, Ze-Zhang Tao

**Affiliations:** 1 Department of Otolaryngology-Head and Neck Surgery, Central Laboratory, Renmin Hospital of Wuhan University, 238 Jie-Fang Road, Wuhan, 430060 Hubei China; 2 Institute of Otolaryngology-Head and Neck Surgery, Renmin Hospital of Wuhan University, 238 Jie-Fang Road, Wuhan, 430060 Hubei China

**Keywords:** Quantum dots, Allergic rhinitis, Immunoglobulin E, Lateral flow immunoassay

## Abstract

The prevalence of allergic rhinitis (AR) is increasing worldwide. However, the current systems used to measure levels of immunoglobulin E (IgE) in sera are associated with several disadvantages that limit their further application. Consequently, there is a need to develop novel highly sensitive strategies that can rapidly detect IgE in a quantitative manner. The development of such systems will significantly enhance our ability to diagnose, treat, and even prevent AR. Herein, we describe our experience of using quantum dot-based lateral flow immunoassay (QD-LFIA), combined with a portable fluorescence immunoassay chip detector (PFICD), to detect serum-specific IgE against *Dermatophagoides pteronyssinus* (Der-p) and *Dermatophagoides farinae* (Der-f), two common mite allergens in China. Our data showed that our system could detect serum-specific levels of IgE against Der-p and Der-f as low as 0.093 IU/mL and 0.087 IU/mL, respectively. We also established a standard curve to determine serum-specific IgE concentrations that correlated well with the clinical BioIC microfluidics system. The sensitivity of our assay was 96.7% for Der-p and 95.5% for Der-f, while the specificity was 87.2% for Der-p and 85.3% for Der-f. Collectively, our results demonstrate that QD-LFIA is a reliable system that could be applied to detect serum-specific IgE in accordance with clinical demands. This QD-LFIA strategy can be applied at home, in hospitals, and in pharmacies, with reduced costs and time requirements when compared with existing techniques. In the future, this system could be developed to detect other types of allergens and in different types of samples (for example, whole blood).

**Electronic supplementary material:**

The online version of this article (10.1007/s00216-020-02422-0) contains supplementary material, which is available to authorized users.

**Graphical abstract:**
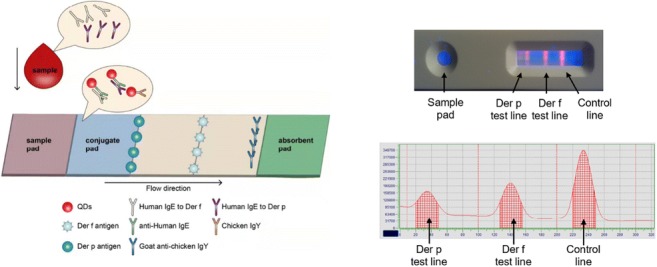
We describe our experiment using a quantum dot-based lateral flow immunoassay combined with a portable fluorescence immunoassay chip detector for both qualitative and quantitative detection of serum-specific IgE against two common mite allergens. This strategy can be applied at home, in hospitals, and in pharmacies, with reduced costs and time requirements. In the future, this system could be developed to detect other types of allergens and in different types of samples.

## Introduction

Between 2005 and 2011, the prevalence of allergic rhinitis (AR) in China increased from 11.1% to 17.6% [1]. Global data also show that almost 40% of the world’s population is now affected by AR [2]. AR has an adverse effect on patients’ quality of life and places a significant burden on their families [3].

AR is characterized by immunoglobulin E (IgE)-mediated inflammation of the nasal mucosa upon exposure to specific allergens. The concentration of total IgE in a healthy adult is approximately 80 kU/L [4, 5]. However, in an allergic scenario, the total IgE level in the blood can increase by four- to 30-fold [6, 7]. Thus, it is evident that IgE plays a fundamental role in the prevention, diagnosis, and treatment of AR. However, both of the currently available and widely established in vivo (e.g., skin prick test, SPT) and in vitro (e.g., the ImmunoCAP system) IgE detection methods are associated with obvious shortfalls which limit their further application. For example, results derived from the SPT can be subjective. To overcome such shortfalls, a range of in vitro assays have been developed, such as the ImmunoCAP system. Although these conventional methods are reasonably accurate and are able to detect IgE, they are not suitable for on-site monitoring, particularly in developing countries with relatively poor access to health facilities. Since demand for such detection systems is increasing, there is a clear need to develop alternative methods for IgE detection that are convenient, sensitive, quantitative, inexpensive, and safe.

Point-of-care testing (POCT) technology has shown excellent potential for the identification of certain disease biomarkers by virtue of the fact that such methods are rapid, simple, efficient, and inexpensive. In recent years, several new strategies have been developed as POCT diagnostic tools [8]. One such strategy is the lateral flow immunoassay (LFIA). LFIA is the combination of a labeled immunoassay with chromatography, in which capillary forces force the analyte to move. Specific recognition elements, representing specific binding moieties, are immobilized on the membrane surface and are thus able to detect different analytes, such as allergens [9, 10]. LFIAs have several key features [11]: (1) reaction speed is fast, occurring in as little as a few minutes; (2) the assay can automatically separate target analytes from biological matrices without complex additional steps; and (3) the assay can be adapted to suit a variety of cluttered outdoor environments, without the need for highly skilled personnel to operate equipment or carry out complex analytical procedures. The first commercial LFIAs were employed for the detection of human chorionic gonadotropin [12]. Since then, the LFIA methodology has attracted significant interest from many disciplines. This is because LFIAs readily meet the ASSURED criteria (Affordable, Sensitive, Specific, User-friendly, Rapid/Robust, Equipment-free and Deliverable to end users) for POCT testing [13]. A variety of LFIAs have now been developed for the detection of chemical contaminants, drugs, biomarkers, toxins, and pathogens, and for disease diagnosis or food analysis [14–18]. However, conventional LFIAs rely on gold nanoparticles as labels and therefore depend upon the localized surface plasmon resonance effect of gold nanoparticles. This only provides qualitative results that are analyzed by the naked eye. Consequently, this labeling system is subjective and inaccurate, and can only be used effectively for the assessment of analytes at high concentrations [19]. Alternatively, organic fluorescent dyes suffer from poor stability, photobleaching, or low quantum yield, thus restricting their widespread application. These issues have led to the development of other labels that might be used to replace gold nanoparticles and thus improve and advance the application of LFIAs, including color latex [20], magnetic nanoparticles [21], and fluorescent reporters [22, 23].

Quantum dots (QDs) [22, 24, 25] are the most promising fluorescent reporters [26, 27] because of their intrinsic properties including high quantum yields, high extinction coefficients, high stability, and long fluorescence lifetimes. Collectively, these properties make QDs an excellent reporter for the development of highly sensitive LFIAs that are capable of quantifying multiple analytes simultaneously. Recent reports have described QD-based LFIAs that employ antigen–antibody reactions to detect the concentrations of a variety of analytes, including tumor markers [28], toxins [29], and viruses [30]. This technology has multiple advantages, including rapid detection, good stability, and low cost; the methodology involved is also user-friendly. A recent publication by Xiong’s group described the development of a size-dependent competitive immunochromatographic assay using QD nanobeads to detect ochratoxin A in corn; this assay exhibited good sensitivity and provided quantitative data [31]. In another study, Wu et al. [32] successfully developed a lateral flow test strip system featuring novel quantum dot-doped polystyrene nanoparticles to detect a cytokeratin-19 fragment and a carcinoembryonic antigen in human serum. More recently, Zhao et al. [33] reported a QD-based lateral flow immunoassay that was used to detect natural product puerarin in both water and biological samples. Although an increasing number of QD-based LFIAs have been reported, this assay has rarely been used in the diagnosis of allergic diseases. It is entirely conceivable that QDs could be conjugated to a targeting anti-human IgE antibody. Thus, we hypothesized that when QDs are functionalized with anti-human IgE antibody, they could be used to detect allergen-specific IgE in serum.

In order to establish this new IgE detection method, we focused initially on the most extensive sources of mite allergens in China [34]: *Dermatophagoides pteronyssinus* (Der-p) and *Dermatophagoides farinae* (Der-f). First, we labeled CdSe/ZnS QDs with anti-human IgE antibody to quantify the specific IgE reaction to Der-p and Der-f in the serum of patients with AR. We then established a standard curve, which showed good correlation with clinical results from the BioIC microfluidic system. We also determined the sensitivity, specificity, detection limit, and reproducibility of our new QD-LFIA strategy; all of these parameters were satisfactory. Our experimental results demonstrated that our QD-LFIAs exhibit good ability to detect IgE in the sera of patients with AR. Future work could lead to the expansion of this technology to a broader range of applications in POCT.

## Methods

### Ethical approval of the study protocol

Our research on human samples was approved by the Ethics Committee of Renmin Hospital within Wuhan University (Wuhan, China). Participants provided written informed consent prior to the study, and all clinical information was anonymized.

### Materials and instruments

Carboxyl-capped fluorescent nanobeads, embedded with CdSe/ZnS QDs, were obtained from Riogene (Beijing, China). Two forms of house dust mites (HDM) were purchased from Stallergenes Greer (London, UK) as a dried powder: natural Der-p allergen (nDer-p) and natural Der-f allergen (nDer-f). Bovine serum albumin (BSA), *N*-(3-dimethylaminopropyl)-*N*′ethylcarbodiimide hydrochloride (EDC), and *N*-hydroxysuccinimide [1-hydroxypyrrolidine-2,5-dione] (NHS), were provided by Sigma-Aldrich (Saint Louis, MO, USA). Antibodies against goat anti-chicken IgY, chicken IgY, and anti-human IgE, were purchased from Abcam (Cambridge, UK). Nitrocellulose (NC) membranes were obtained from Pall Corporation (New York, NY, USA). Semi-rigid polyvinyl chloride (PVC) sheets and the glass fiber used for conjugate pads, sample pads, and absorbent pads were supplied by Shanghai JieYi Biotechnology (Shanghai, China).

Absorption spectroscopy was carried out with an ultraviolet-visible (UV-Vis) spectrophotometer (UV-2450; Shimadzu, Kyoto, Japan). Fluorescence spectroscopy was undertaken on a fluorescence spectrometer (LS-55; PerkinElmer, Waltham, MA, USA). The morphology and size of the QDs were analyzed by transmission electron microscopy (TEM) using an H-7650 (Hitachi, Tokyo, Japan) system.

### Preparation of QD–antibody conjugates

The process of conjugation between a selected antibody and the QDs took place in a dark environment; this ensured that the QDs could avoid fluorescence quenching in the presence of light. The antibodies to be labeled were fully dialyzed against phosphate-buffered saline (PBS; 50 mM; pH 7.2). If the antibody concentration was low, we carried out a concentration protocol until the concentration was >5 mg/mL, actually was 10mg/ml. Absorbing 100 ml QD solution (1.2 mg/mL), successively adding 0.2 mg NHS (2 mg/ml), which was prepared with 50 mM morpholine ethanesulfonic acid (MES) (pH 5.5) buffer solution, and 0.3 mg EDC (3 mg/ml), which was prepared with 50 mM MES (pH 5.5) buffer solution, to a clean eppendorf tube. The mixture was mixed using a magnetic stirrer for 30 min at 37 °C. After centrifugation at 10,000×*g* for 15 min at room temperature, the supernatant was removed, and the microspheres were washed twice with MES buffer (pH 5.5). Next, the dialyzed antibody (400 g) was added, agitated, and mixed evenly at 60 rpm for 2 h at 37 °C. This was followed by another centrifugation step at 10,000×*g* for 15 min at room temperature. The supernatant was then removed, and 100 μL of 2 M glycine was added. The mixture was agitated and mixed evenly for 30 min at 37 °C. If agglomeration occurred, the supernatant was dispersed and blended by ultrasound. Finally, the supernatant was centrifuged at 10,000×*g* for 15 min at room temperature, the supernatant removed, and 50 mM of BSA (0.5%) added. The volume was then made up to 500 mL (if necessary, ultrasound was employed for 30 s) and stored at 4 °C to await future application [35].

### Fabrication of lateral flow test strips

**Fig. 1:**
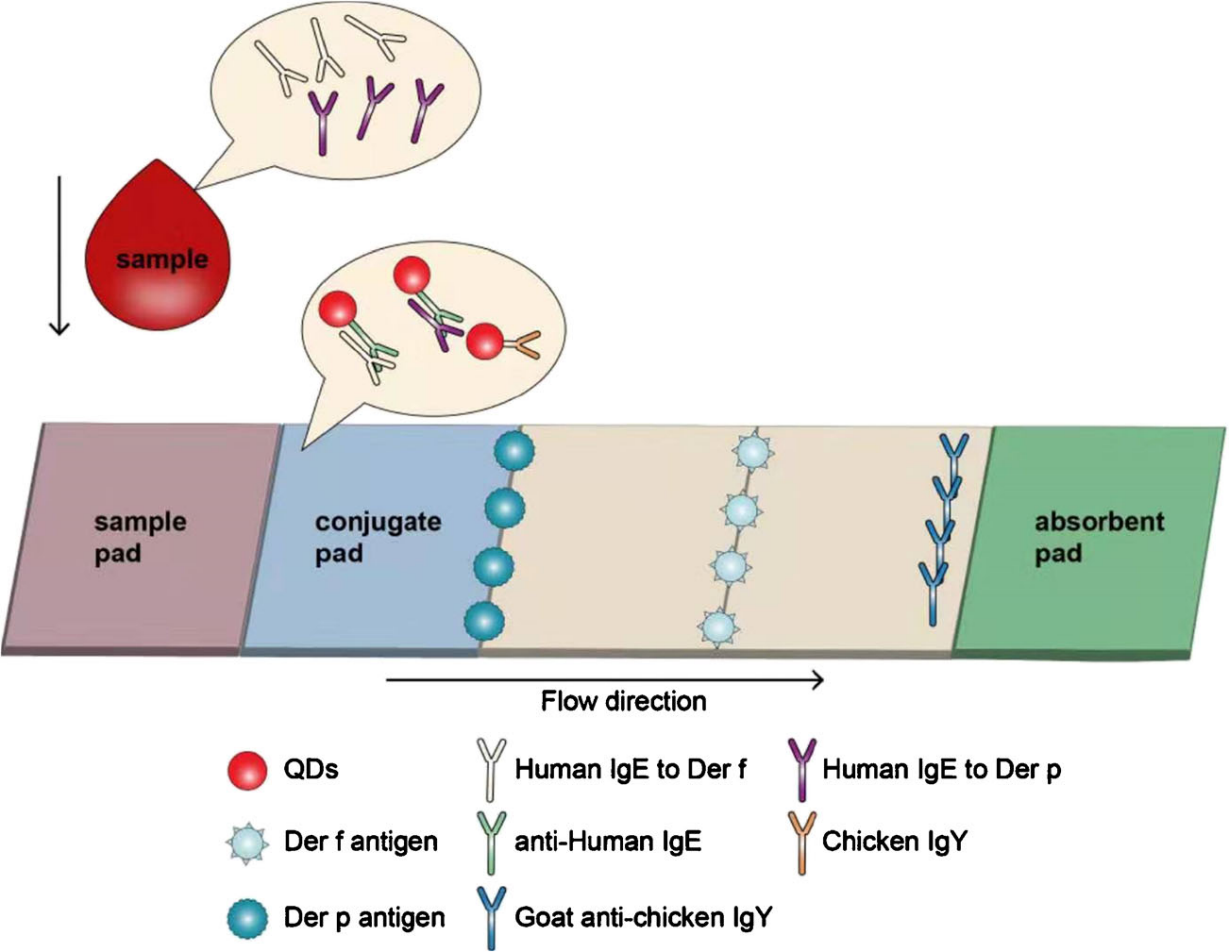
Schematic of QD-based LFIA. Serum sample containing specific IgE was dropped onto the sample pad and migrated along the strip. First, specific IgE combined with QDs labeled with anti-human IgE in the conjugate pad. The formed complexes continued to migrate along the membrane and were captured by Der-p/Der-f immobilized on test lines and formed QD-labeled anti-human IgE–specific IgE–Der-p/Der-f complexes. As the liquid continued to migrate, residual QD-labeled chicken IgY was captured by goat anti-chicken IgY immobilized on the last control line. Excess QD conjugates continued to migrate towards the absorbent pad

The main components of the lateral flow test strips (sample pad, NC membrane, and an absorbent pad) were assembled on a PVC backing pad (Fig. 1). First, the NC membrane was laminated and adhered to a plastic backing sheet. The test line was then prepared by dispersing Der-p and Der-f proteins in buffer solution. Goat anti-chicken IgY was immobilized (at a concentration of 1 mg/mL) as the control line. After protein immobilization, the NC membrane was dried for 24 h at 47 °C and then blocked with PBS containing 1% BSA. The sample pad and absorbent pad were laminated sequentially and adhered on the backing sheet with overlaps to ensure that the test solution could migrate through the test strip. The entire assembled card was cut into strips 4 mm in width, and assembled into the plastic card. The strips were then stored at room temperature in a sealed bag with desiccant.

### Principles underlying the detection and testing procedure

To carry out the fluorescence immunoassay, 20 μL of patient serum was added to 100 μL of buffer solution. After thorough mixing, 100 μL of the fluorescent solution was dropped onto the sample hole and allowed to react for 15 min. We then acquired fluorescence images of the lateral flow strips under UV-light illumination with a digital camera (G7 X; Canon, Tokyo, Japan). Quantitative analyses of fluorescence were performed using a portable fluorescence immunoassay chip detector (PFICD).

### Serum collection

Venous blood samples (5 mL) were collected from 61 patients with Der-p-positive AR and 68 patients with Der-f-positive AR; all patients fasted overnight at Renmin Hospital prior to providing blood samples. We ensured that the study population had never received specific immunotherapy against Der-p or Der-f. Serum was subsequently separated from each sample and stored at −20 °C to await further analyses. Serum samples were mixed thoroughly after thawing to ensure consistency; repeated freezing and thawing was avoided. The control groups included 39 individuals without allergy to Der-p and 32 individuals without allergy to Der-f.

## Results

### Characterization of QDs

**Fig. 2:**
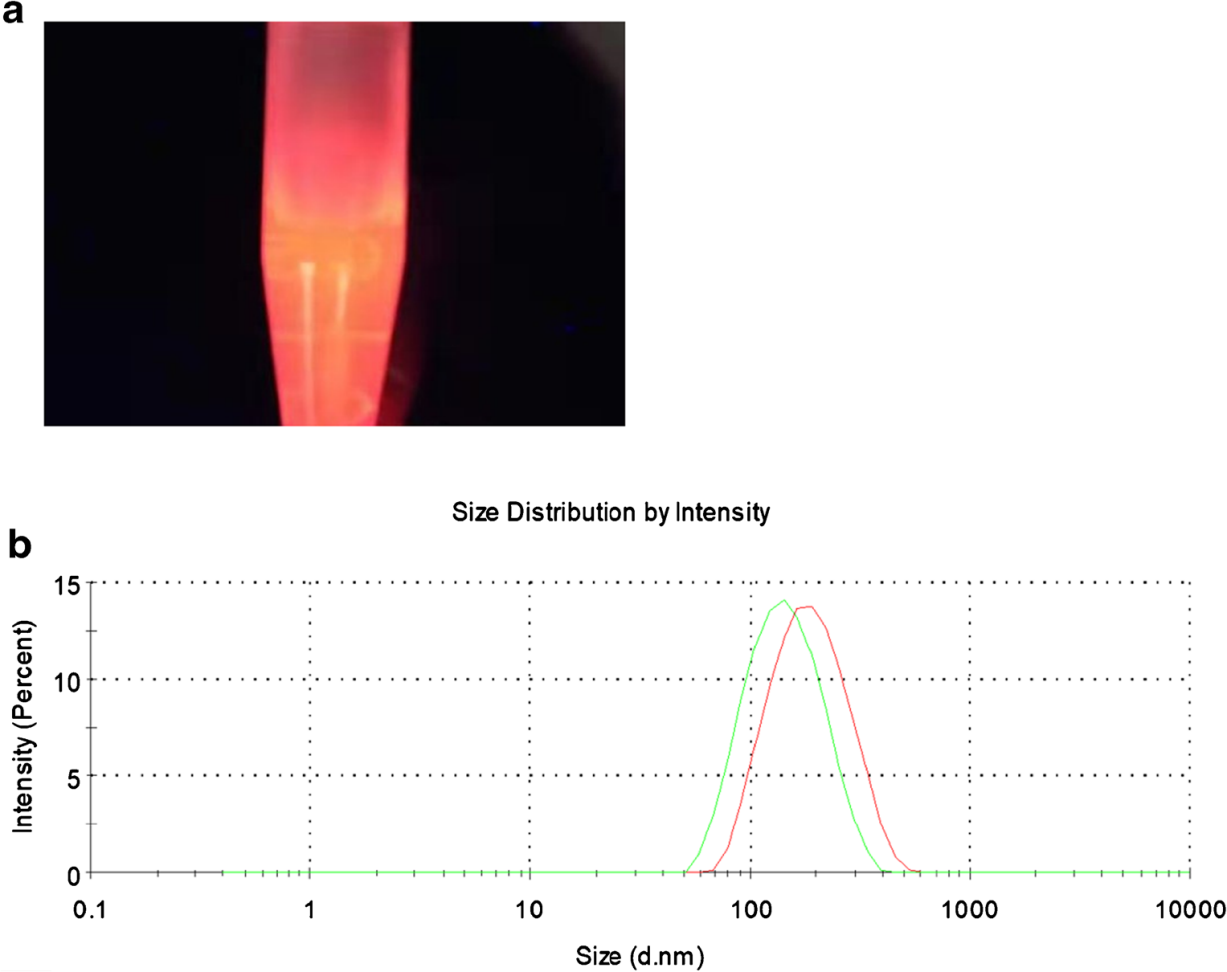
Characterization of QDs: excitement in UV light (**a**) and size distribution of QDs and QD-anti human IgE conjugate analyzed by dynamic light scattering (**b**)

Steady, bright orange fluorescence images of “naked” QDs excited by UV light are shown in Fig. 2a. We characterized QDs by TEM; see Electronic Supplementary Material (ESM) Fig. S1). On TEM images, QDs appeared as dark dots, presented in the form of composite nanobeads, and could be readily identified from the polymer matrix due to differences in electron penetrability. The size of the QD microspheres, as calculated from TEM images, was approximately 100 nm, which was slightly smaller than the size of 150 nm measured by dynamic light scattering (Fig. 2b). Furthermore, the crystal lattice of the nanocrystals observed in the dark dots confirmed that QDs were enclosed in the polymer nanobeads.

### Emission and excitation of the QD and QD–antibody conjugates

**Fig. 3:**
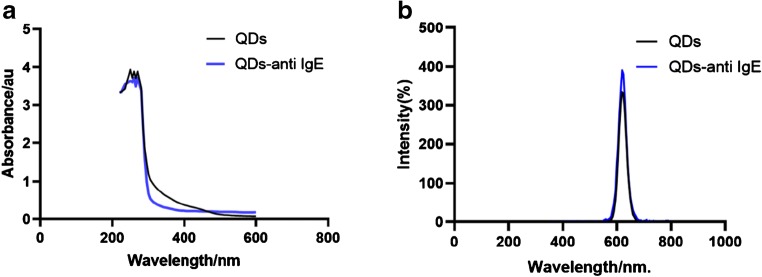
Emission and excitation of naked QDs and QD-labeled anti-human IgE **a** Naked QDs and QD-labeled anti-human IgE were excited from 200 nm to 600 nm. **b** A distinct small peak at ~280 nm was observed. The emission peaks of QDs and QD-labeled anti-human IgE were formed at 620 nm. The results of two independent experiments showing a similar trend are depicted

Anti-human IgE antibodies were covalently conjugated with QDs by coupling the carboxyl groups located on the surface of the QDs and the amino groups exiting the antibody molecule; this was accomplished by carbodiimide chemistry [36]. To confirm that conjugation was successful, we acquired UV-Vis absorbance spectra in order to compare the naked QDs with the QD–antibody conjugates. First, we measured their absorbance using a continuous range of light excitation from 200 nm to 600 nm. QD-labeled anti-human IgE showed a distinct absorbance peak at ~280 nm, indicating that antibody was present (280 nm is a characteristic peak for proteins). Furthermore, the manner of excitation was similar to that of naked QDs (Fig. 3a). We also used a spectrophotometer to measure emitted light. QD-labeled anti-human IgE exhibited a strong fluorescence signal with narrow emission spectra. This was similar to that of naked QDs, and the emission peak was ~620 nm, which was similar to the emission peak of naked QDs (Fig. 3b). In other words, our QD-labeled anti-human IgE complex retained the characteristic features of QDs and antibodies.

### Detection of specific IgE using QD-based LFIA

Serum samples were mixed with functionalized QDs, dropped onto the sample pad, and moved forward along the LFIA via capillary action (Fig. 1). First, specific IgE in the serum samples combined with QDs that had been labeled with anti-human IgE on the conjugate pad. The formed complex then reached the test lines and was captured by the allergen immobilized on the NC membrane to form QD-anti-human IgE–specific IgE–Der-p/Der-f sandwich complexes. As the liquid sample continued to migrate forward, the residual QD-labeled chicken IgY was captured by the goat anti-chicken IgY that was immobilized on the control line. After these two reactions, functionalized QDs were immobilized at the test lines and the control line; in contrast, excess QDs progressed along the membrane to the absorbent pad. This process lasted 15 min. Subsequently, a fluorescent signal could be observed under UV light by the naked eye. This permitted a qualitative yes/no result; quantitative results could also be obtained using a normal PFICD.

### Testing serum samples

We collected serum samples with known specific IgE concentrations, classified into seven clinical stages (Table 1), from patients who were Der-p-positive (*n* = 61), Der-f-positive (*n* = 68), Der-p-negative (*n* = 39), and Der-f-negative (*n* = 32). These samples were all analyzed by the BioIC microfluidic system and our QD-LFIA; these techniques allowed us to establish a standard curve and determine the detection limit, sensitivity, and specificity.

**Table 1:** Specific IgE level tested by the BioIC microfluidic system

IU/ml	Level
<0.35	0
0.35–0.7	1
0.7–7.7	2
7.7–21.8	3
21.8–50	4
50–100	5
50–100	6

**Fig. 4:**
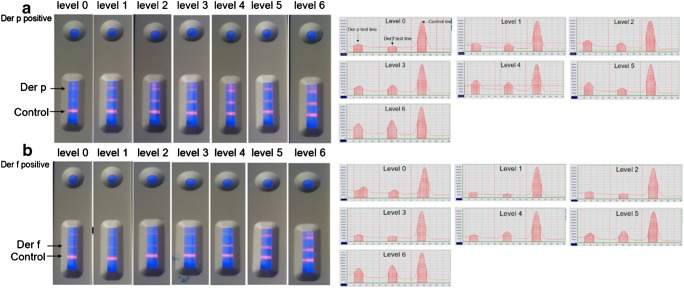
Assay results using the naked eye and PFICD. **a** Levels of allergy to Der-p (Table 1) in order; “a” indicates the test line (T) immobilized with Der-p antigen, while “c” indicates the control line (C) immobilized with goat anti-chicken IgY. The signal curve on the right represents the quantitative results obtained by the PFICD, and we established the standard curve for the Der-p allergen based on the T/C ratio. **b** Levels of allergy to Der-f (Table 1) in order: “b” indicates the test line immobilized with Der-f antigen, while “c” indicates the control line immobilized with goat anti-chicken IgY. The signal curve on the right represents the quantitative results obtained by the PFICD, and we established the standard curve for the Der-f allergen based on the T/C ratio

Because of the efficient fluorescence and photostability of our QDs, fluorescence images on the test and control lines on the test strip could be readily observed by the naked eye upon excitement by an ultraviolet lamp; this permitted a simple “yes/no” answer. We identified a dose-dependent relationship between the fluorescence intensity and concentration of specific IgE (Fig. 4); we expected this because higher concentrations of specific IgE led to the formation of a greater number of sandwich complexes. Furthermore, the control line was readily observed in the absence or presence of both Der-p and Der-f allergens.

**Fig. 5:**
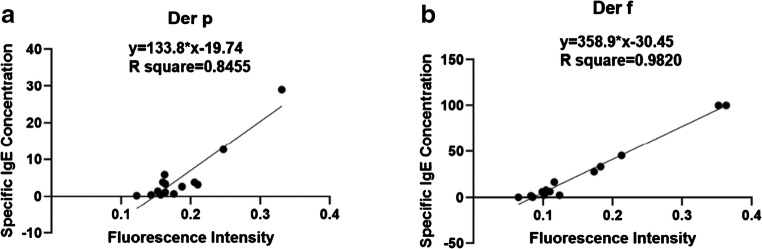
Standard curve for fluorescence intensity vs. specific IgE concentration. Standard curves were constructed based on measurements of a series of known specific IgE concentrations determined by the BioIC microfluidic system. The best-fit calibration was described by *y* = 133.8 × *x* − 19.74 (*R*^2^ = 0.8455) for Der-p and *y* = 358.9 × *x* − 30.45 (*R*^2^ = 0.9820) for Der-f

To obtain quantitative results, we used a normal PFICD. Standard curves were constructed based on clinical samples with known specific IgE concentrations, as determined by the clinical BioIC microfluidic system. We demonstrated that the ratio of fluorescence intensity between the test line and the control line (T/C) increased with increasing concentrations of specific IgE. The best-fit calibration equation for Der-p was *y* = 133.8 × *x* − 19.74 (*R*^2^ = 0.8455), while that for Der-f was *y* = 358.9 × *x* − 30.45 (*R*^2^ = 0.9820) (Fig. 5). Therefore, once the T/C value was calculated from the PFICD, it was possible to determine unknown IgE concentrations in serum samples from patients.

**Fig. 6:**
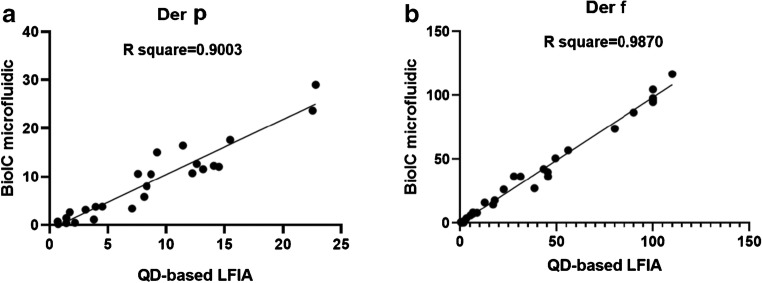
Correlation between the BioIC microfluidic method and QD-based LFIA. We compared the quantitative specific IgE results of our QD-based LFIA obtained on the basis of the standard curve established above with clinical results, which were in good accordance with each other

We also compared these quantitative results with data generated by the clinical BioIC microfluidic system, which showed that the two sets of data were in good agreement (Fig. 6). Analysis of the negative serum samples was carried out 20 times in order to generate a mean fluorescence value and thus determine the limit of detection (see ESM Table S1). Our QD-LFIA assay detected specific IgE levels to Der-p that were as low as 0.093 IU/mL, and as low as 0.087 IU/mL for Der-f. Generally, specific IgE levels > 0.35 IU/mL can be detected by current in vitro technologies. Consequently, our QD-based LFIA could be used for clinical application.

Finally, we determined the specificity and sensitivity of our new assay. We observed sensitivity of 96.7% and 95.5% for Der-p and Der-f, respectively (Table 2), and specificity of 87.2% for Der-p and 85.3% for Der-f (Table 2), thus linking QD-LFIA data from the PFICD. We also prepared and maintained QD-LFIA test strips at 4 °C for 1 year and examined the fluorescence signals once every 2 weeks. No obvious difference was observed during this period, which suggested that these products exhibited stable fluorescent signals and good reproducibility.

**Table 2:** Serum test results for human IgE to Der-p or Der-f obtained by QD-based LFIA compared with that obtained by the BioIC microfluidic system

BioIC microfluidic	QD-based LFIA		Total number
	Positive (Der-p/Der-f)	Negative (Der-p/Der-f)	(Der-p/Der-f)
Positive	59/63	2/5	61/68
Negative	5/3	34/29	39/32
Total number	64/66	36/34	100/100

## Discussion

In recent years, POCT has been widely applied for clinical diagnostics, environmental monitoring, and food analysis. LFIA is a form of POCT that has been applied to several aspects of immunological diagnostics [37–44]. Previous studies involving the detection of allergens were mostly limited to qualitative or semiquantitative analysis, which led to the development of QDs in an attempt to improve sensitivity and provide quantitative analysis [4, 45–47]. However, previous studies largely ignored the possibility of using this promising strategy to characterize allergic disease. In view of this, we attempted to establish a QD-LFIA platform that was able to detect specific IgE in allergic disease. We anticipated that such a system would be superior to traditional detection methods such as the skin prick test (in vivo), the BioIC microfluidic chip system (in vitro), or the ImmunoCAP system (in vitro).

The skin prick test (SPT) has been widely applied for the detection of allergic disease, largely because it is rapid and inexpensive, and exhibits high sensitivity. Our QD-LFIA strategy can be completed as rapidly as the SPT, in only 15 min, and following commercialization should be available at low cost. On the other hand, the SPT works better when carried out by professional staff in a controlled laboratory, when patients have no obvious allergic systems, and when patients have not been administered antihistamines or corticosteroids. In patients with serious allergies, SPT may lead to strong local reactions, and even anaphylactic shock. Consequently, subjects receiving the SPT should be monitored carefully for several hours. Moreover, results from the SPT are determined according to wheal size; this depends on observations by a specialist. With our new QD-LFIA strategy, it is possible to acquire sensitive, quantitative results without strict demands on the patient (serious or minor allergy, medication use). Moreover, there is no need for a specialist or professional laboratory, and there is little or no risk of adverse accidents (Table 3).

**Table 3:** Comparison of allergen detection methods

Methods	Advantages	Shortcomings
Skin prick test (SPT)	-The best in vivo test -Simple operation -High sensitivity -Low cost -Highly relevant for clinical setting	-Influenced by many factors (e.g., drug) -Results often determined visually -Need professional staff to operate
ImmunoCAP	-Fewer influence factors -High sensitivity -Automated operation	-Time cost -High cost
QD-LFIA	-Fewer influence factors -High sensitivity and specificity -Automated operation -Fast results -Low cost -Not only qualitative but also quantitative results -Easy to collect sample materials	-Technology needs to be improved

The currently available in vitro specific IgE detection techniques, such as the ImunnoCAP system, are minimally invasive, safe, and automated. However, in most cases, such assays take more than 1 h to complete and incur significant costs, thus causing economic burden to poor and non-local patients. Furthermore, the detection limits of these techniques are normally around 0.35 IU/ml. Consequently, these assays could ignore meaningful results that fall below the detection limit, leading to false-positive results. Finally, the reproducibility and accuracy of these techniques is not sufficient (Table 3).

A previous study managed to partially overcome such limitations by establishing a QD-LFIA method that was combined with image analysis for the detection of specific IgE to Der-p [36]. The detection limit for this assay was 0.2 IU/ml, thus representing an improvement over the 0.35 IU/ml limit of the more conventional systems. The system also produced semiquantitative data by utilizing a digital camera and ImageJ software. In the present study, we describe a new assay that achieves a further improvement in the detection limit, from 0.2 IU/ml to 0.093 IU/mL for Der-p, and to 0.087 IU/mL for Der-f. Moreover, our assay yielded quantitative results, and used a normal PFICD that was simpler to use than the image analysis software described in the earlier study. We also focused on two different mite allergens, Der-p and Der-f, so that we could establish two test lines in a single strip. While we were successful, further research is now needed to improve the sensitivity (96.7% for Der-p and 95.5% for Der-f) and specificity (87.2% for Der-p and 85.3% for Der-f) of the system. We prefer to use QD nanobeads for antibody conjugation; we do this because the ratio of QD-to-antibody can be significantly increased by the encapsulation of many QDs in one nanobead, thus leading to increased sensitivity for the QD–antibody conjugates. We tested two different aperture sizes (15 μm and 8 μm), as different aperture sizes show different flow speeds. A high flow speed would reduce sensitivity, while a low flow speed would increase the risk of false-positive data. We found that a low flow speed worked better. We also investigated the optimal reaction conditions, including immobilization concentration, pH, temperature, and electrolytes. We diluted allergens to 0.5, 1.0, 1.5, and 2.0 mg/mL (see ESM Fig. S2), and selected a dilution of 1.5 mg/ml for further analysis, because this concentration showed the best linear correlation with fluorescence intensity. We tested three types of buffer solution (see ESM Fig. S3) in order to optimize activity and stability upon allergen dilution. We also tested three types of anti-human IgE (see ESM Fig. S4) in order to optimize activity and specificity. We chose goat anti-chicken IgY as the control line to reduce possible cross-reactions in the presence of IgE. Collectively, this approach led to the optimization of methods and materials to achieve the most reliable, sensitive, and quantitative results.

Our research showed that the new QD-LFIA provides a qualitative “yes/no” answer by the naked eye, but also precise quantitative results generated from a normal PFICD. This QD-LFIA platform could be applied not only in hospitals, but also in the home or pharmacy; the technology would therefore suit different people with different needs. However, samples that were positive for other allergens, such as artemisia or milk, are too rare to collect in our area. These could be included in future large-scale studies that aim to evaluate the QD-LFIA platform for other allergens. We also believe that this QD-LFIA strategy could be employed to assay samples of whole blood (or even nasal mucus), and therefore provide a fast, efficient, and accurate system for the future.

## Conclusions

We aimed to develop a convenient and sensitive strategy for detecting specific IgE in serum. We successfully established a QD-LFIA system that was simple to use and provided sensitive quantitative results in detecting specific IgE to Der-p and Der-f allergens in serum samples, with remarkably low detection limits and high levels of both sensitivity and specificity. This platform could be applied to detect other allergens and with other sample sources in the future, and could therefore transform the diagnosis of AR.

## Electronic supplementary material

ESM 1 (PDF 444 kb)
