# Supplementary material for: A quantum dot-based lateral flow immunoassay for the rapid, quantitative, and sensitive detection of specific IgE for mite allergens in sera from patients with allergic rhinitis
Source: Anal Bioanal Chem. 2020 Feb 12;412(8):1785–94. doi: 10.1007/s00216-020-02422-0 (PMC7048869; doi:10.1007/s00216-020-02422-0)
Supplement: Supplementary file 1 — (PDF 444 kb) [file 216_2020_2422_MOESM1_ESM.pdf]

Analytical and Bioanalytical Chemistry

Electronic Supplementary Material

A quantum dot-based lateral flow immunoassay for the rapid, quantitative, and sensitive detection of specific IgE for mite allergens in sera from patients with allergic rhinitis

Zheng-Yan Liang, Yu-Qin Deng, Ze-Zhang Tao

Table S1. Detection limit of serum specific IgE detected by QD-LFIA method

Der p negative samples	T/C	Der f negative samples	T/C
1	0.0038	1	0.0115
2	0.0033	2	0.0097
3	0.0003	3	0.0084
4	0.0036	4	0
5	0.0061	5	0.0114
6	0.0026	6	0
7	0.0046	7	0.0069
8	0.0046	8	0.0101
9	0.0053	9	0
10	0.0024	10	0.0118
11	0.002	11	0.0046
12	0.0043	12	0.0115
13	0.0049	13	0.0029
14	0.0017	14	0.0106
15	0.0025	15	0.0101
16	0.0038	16	0.0068
17	0.0027	17	0.0001
18	0.004	18	0.0079
19	0.004	19	0.0006
20	0.0002	20	0.0125
M	0.0033	M	0.0069
SD	0.0015	SD	0.0047

M+2SD	0.0064	M+2SD	0.0162
Detection limit	0.093	Detection limit	0.087

M: mean; SD: standard deviation

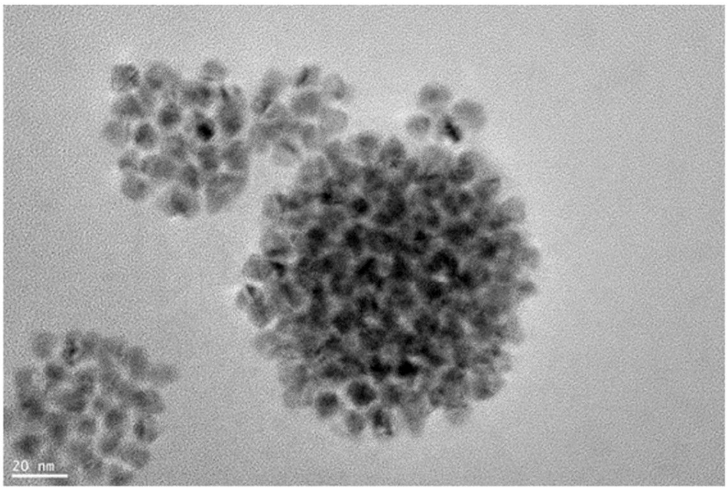

Figure S1. Transmission electron microscope (TEM) image of naked QDs

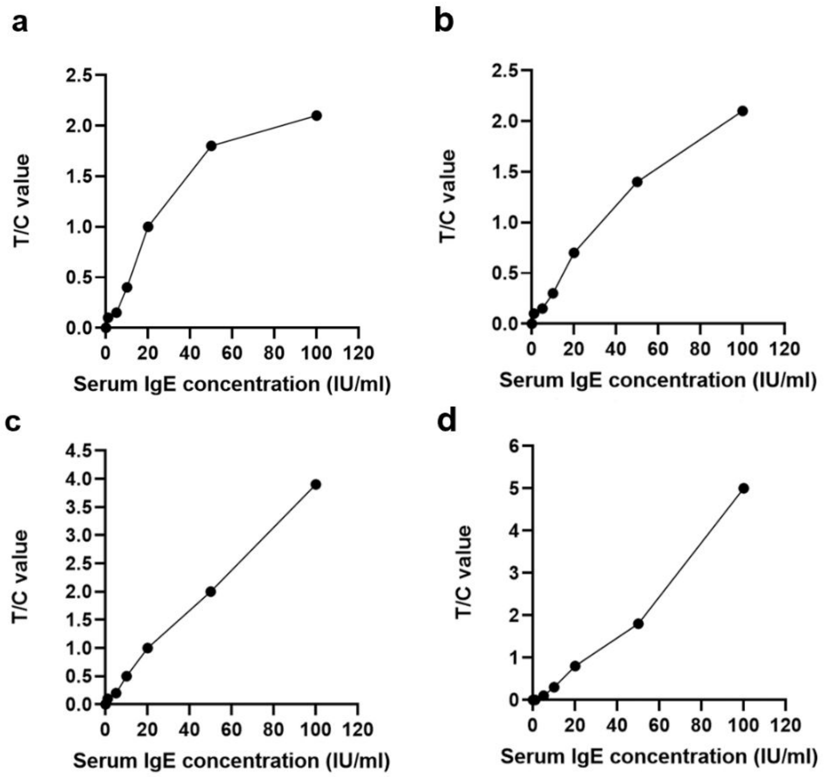

Figure S2. Testing different antigen concentration coated on the pad

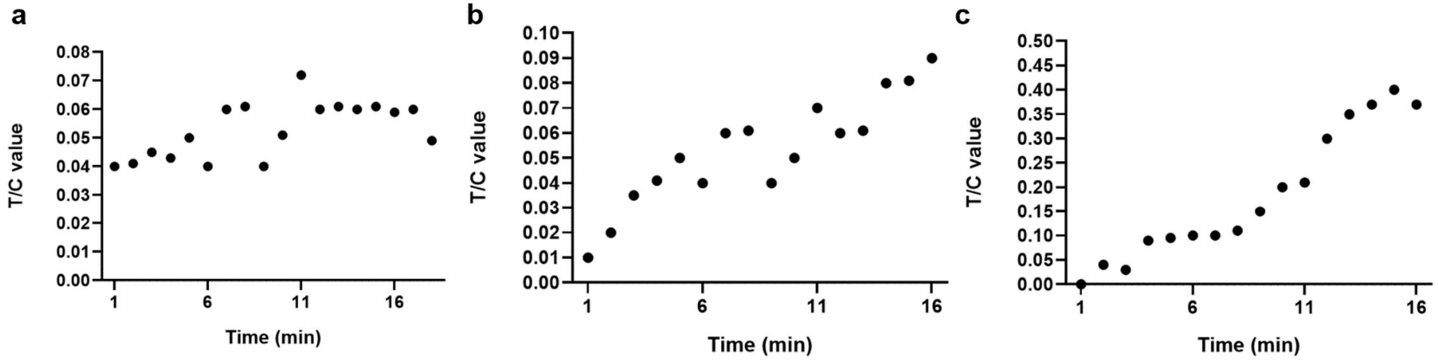

Figure S3. Testing different coated buffer solutions
